# Chronic and Atypical Presentation of Superior Vena Cava Syndrome: A Case Report

**DOI:** 10.7759/cureus.94283

**Published:** 2025-10-10

**Authors:** Daniel Rosales Rosales, Carlos A Jaimes Loreto, Quitzia L Torres Salazar

**Affiliations:** 1 Internal Medicine, Universidad Juárez del Estado de Durango, Durango, MEX; 2 Internal Medicine, Universidad Autónoma de Durango, Durango, MEX; 3 Biomedical Sciences, Universidad Juárez del Estado de Durango, Durango, MEX

**Keywords:** adenocarcinoma, case report, collateral venous circulation, mediastinal mass, superior vena cava syndrome

## Abstract

We report the case of a 66-year-old woman with an unusually chronic presentation of superior vena cava syndrome (SVCS), characterized by more than two decades of collateral venous development before definitive evaluation. The patient presented with a 10-month history of persistent dry cough and long-standing venous engorgement of the supraclavicular, thoracic, and abdominal regions, which she recalled dating back to the mid-1990s. Despite the extent of venous changes, she denied constitutional symptoms, respiratory distress, or neurological compromise. Physical examination confirmed prominent collateral circulation, tracheal deviation, and absent breath sounds in the right apex. Laboratory investigations were within normal limits except for mild anemia and dyslipidemia. Computed tomography of the chest revealed a large bilobulated mediastinal mass, measuring 70 × 67 × 121 mm, compressing the superior vena cava and the brachiocephalic trunk, with associated venous dilatation. Differential diagnosis included thymoma, lymphoma, and germ cell tumor. Ultrasound-guided biopsy demonstrated malignant epithelial cells with tubulo-glandular architecture, consistent with adenocarcinoma. Unlike most malignant SVCS cases, which present acutely and demand urgent management, this patient remained clinically compensated for decades, highlighting the remarkable capacity of venous adaptation. The protracted course allowed a complete diagnostic workup and histopathological confirmation before therapeutic decisions were required. This report underscores the importance of recognizing collateral venous networks as a clinical sign of central venous obstruction, even in the absence of acute symptoms. It also illustrates the relevance of physical examination in guiding diagnosis in an era dominated by imaging. Awareness of such atypical and indolent cases can help clinicians avoid delayed recognition and ensure timely oncologic management.

## Introduction

Superior vena cava syndrome (SVCS) is a clinical condition resulting from impaired venous return from the head, neck, and upper extremities due to obstruction of the superior vena cava. The syndrome is most frequently associated with malignant etiologies, primarily lung cancer and lymphomas, which account for approximately 70% of cases [1]. Benign causes represent about 30% of cases, with an increasing proportion related to intravascular devices such as central venous catheters and pacemaker leads [2].

Clinically, SVCS often presents with an acute or subacute onset of symptoms such as facial and neck edema, dyspnea, distended thoracic veins, and cough, which occur in 60-100% of patients depending on the severity of obstruction. In life-threatening cases, rapidly progressive obstruction can cause cerebral or laryngeal edema, whereas the establishment of collateral venous pathways may alleviate hemodynamic compromise and attenuate symptoms [1].

Chronic or indolent presentations of SVCS are rare. Slow-growing mediastinal tumors or benign fibrosing processes may allow gradual development of collateral circulation, permitting long-term compensation and delaying clinical recognition. In such scenarios, patients can remain stable for years despite extensive obstruction, and superficial collateral veins may be the only overt clinical manifestation [3]. Reports of SVCS with decades-long evolution before diagnosis are exceptionally uncommon in the literature.

Management strategies for SVCS have evolved from urgent radiotherapy to multidisciplinary approaches incorporating endovascular stenting, chemotherapy, and surgery. Endovascular therapy has become the preferred first-line treatment in many centers, achieving rapid symptom relief in over 90% of cases. Nonetheless, chronic cases such as the one reported here highlight diagnostic challenges, the potential for misinterpretation, and the continued importance of careful physical examination in an era dominated by advanced imaging [4].

In this report, we describe a case of SVCS secondary to a mediastinal mass ultimately diagnosed as adenocarcinoma, with an unusually long clinical course extending for more than two decades before definitive evaluation. This case illustrates both the adaptive capacity of the venous system and the importance of recognizing atypical features of SVCS. Recent clinical observations have emphasized the relevance of chronic or slowly progressive forms of SVCS with extensive collateral development. Black et al. reported a case of lung adenocarcinoma presenting predominantly with distended superficial thoraco-abdominal veins and minimal acute symptoms, in which computed tomography demonstrated marked collateral vascularization secondary to superior vena cava obstruction. Their report illustrates how gradual venous blockade can allow compensatory hemodynamic adaptation, resulting in a deceptively stable presentation. Including such cases provides a concrete reference point for comparing the present report and underscores the diagnostic value of recognizing chronic collateral circulation patterns [3]. This case report has been prepared in line with the SCARE 2025 criteria [5].

## Case presentation

A 66-year-old woman presented to the outpatient clinic for evaluation of a persistent dry cough of 10 months’ duration. The cough occurred in paroxysmal episodes without identifiable attenuating or exacerbating factors and was not accompanied by dyspnea, cyanosis, or systemic manifestations. On directed questioning, she reported the insidious appearance of dilated superficial veins over the supraclavicular, thoracic, and abdominal regions, which she had first noticed as early as 1995. Despite the chronicity of these findings, she had not pursued medical evaluation at that time and denied constitutional symptoms, including fever, night sweats, or significant unintentional weight loss. She expressed concern about the aesthetic changes in her chest and abdominal wall but reported that these had not interfered with her daily functional capacity (Table 1).

**Table 1:** Clinical timeline Clinical timeline summarizing the prolonged evolution of chronic superior vena cava syndrome in the present case. SVC: Superior vena cava

Year/Period	Clinical Event or Observation	Clinical Significance/Notes
~1995	The patient first noticed mild venous engorgement over the anterior chest wall.	Initial manifestation of slowly developing collateral circulation; no diagnostic work-up performed.
2008	Diagnosis of type 2 diabetes mellitus.	No direct relation to venous symptoms; indicates onset of comorbid metabolic disease.
2015	Diagnosis of hypertension.	Continued stable course; no respiratory or facial symptoms reported.
2023–2024	Gradual appearance of visible abdominal wall veins and intermittent dry cough.	Suggestive of progressive but compensated obstruction.
Early 2025	Evaluation for chronic cough → Chest CT revealed a mediastinal mass compressing the SVC with extensive collateral formation.	Imaging confirmation of chronic SVC obstruction.
2025	Ultrasound-guided biopsy → Histology consistent with adenocarcinoma.	Etiologic diagnosis established; the patient remained hemodynamically stable.

Her past medical history was significant for type 2 diabetes mellitus, diagnosed in 2008, managed with dapagliflozin 10 mg orally once daily and insulin glargine 20 IU administered subcutaneously once daily. She had arterial hypertension since 2015, treated with irbesartan 150 mg orally once daily, bisoprolol 5 mg orally once daily, and amlodipine 5 mg orally twice daily. Additionally, she reported chronic intake of rivaroxaban 10 mg, at a quarter of a tablet once daily, for unclear indication. She denied any prior hospitalizations or surgeries. There were no known drug allergies. Family history was noncontributory, with no reported inheritable conditions. Socially, the patient was a retired homemaker, nonsmoker, with no history of alcohol or recreational drug use, living independently with her spouse.

On review of systems, she denied neurological symptoms such as headache or visual disturbances, as well as cardiovascular symptoms, including chest pain or palpitations. She reported no gastrointestinal complaints beyond abdominal distension related to prominent venous collaterals and denied urinary or musculoskeletal symptoms.

**Figure 1:**
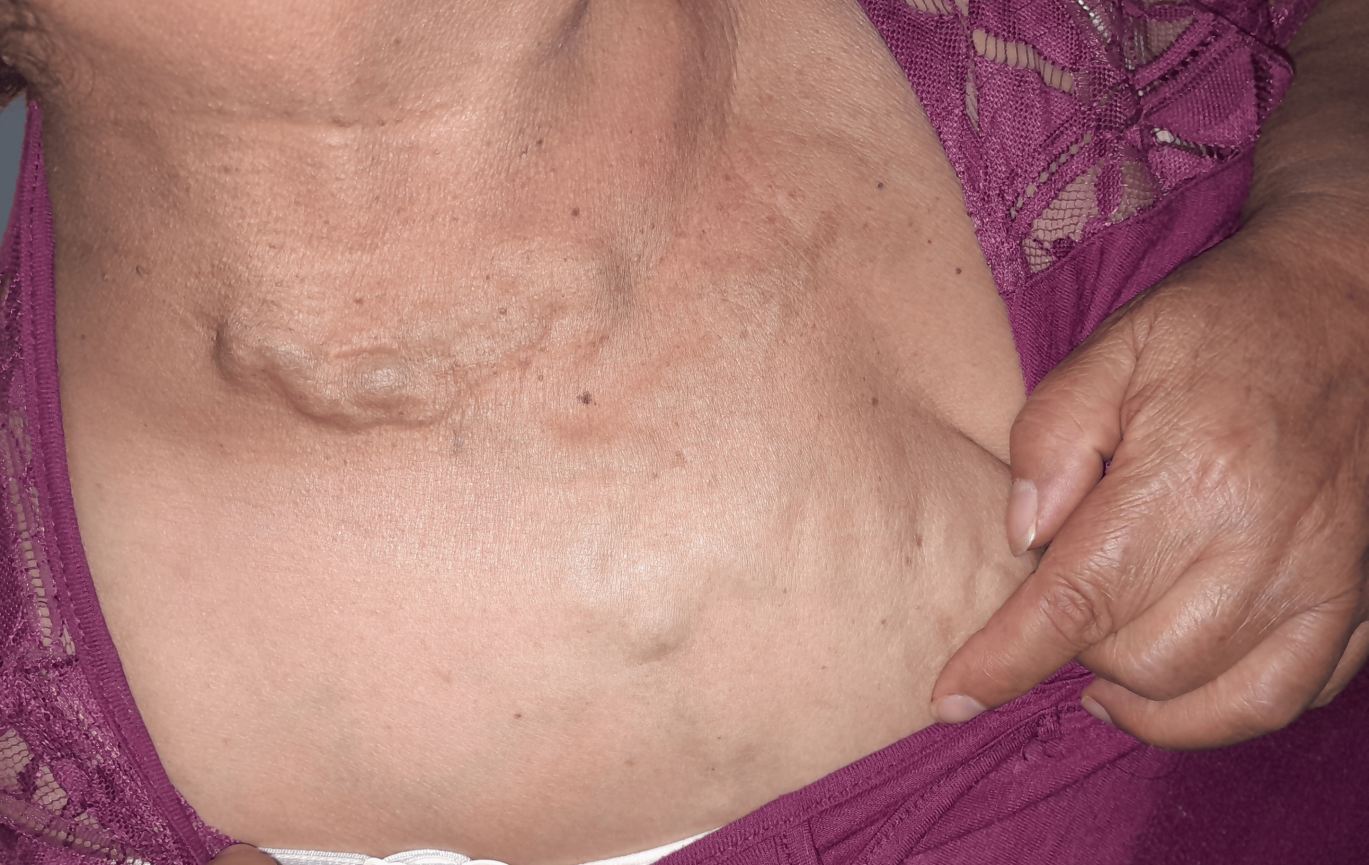
Clinical photograph showing prominent collateral venous circulation over the anterior chest and supraclavicular region, consistent with chronic superior vena cava obstruction

**Figure 2:**
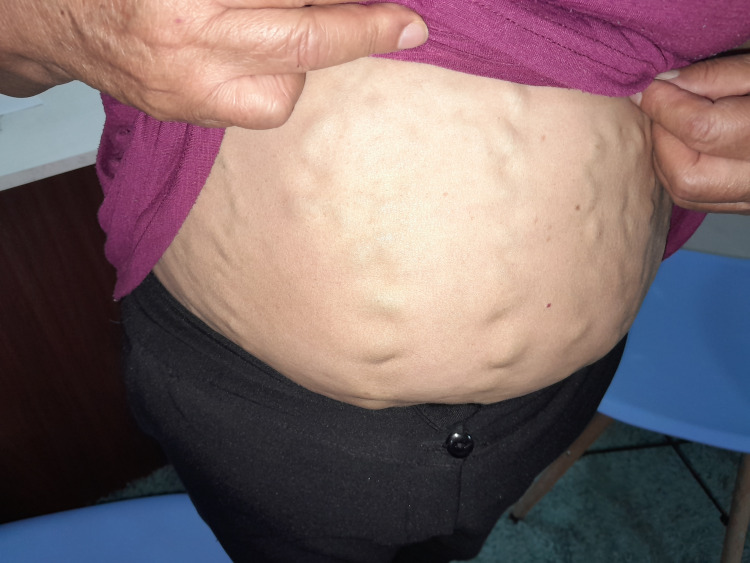
Prominent collateral venous circulation over the anterior abdominal wall, reflecting long-standing superior vena cava obstruction with extensive collateral network development

On physical examination, the patient was alert and in good general condition. The neck was symmetrical with slight left tracheal deviation. Prominent collateral veins were observed over the anterior chest and abdominal wall (Figures 1, 2). On bedside compression-refill assessment, abdominal wall collaterals filled in a cranio-caudal direction, indicating diversion toward the inferior epigastric and iliac venous systems, consistent with the physiologic pattern of chronic superior vena cava obstruction. Auscultation revealed absent vesicular breath sounds in the right apex, while the remainder of the lung fields were preserved. Cardiac evaluation showed regular rhythm and normal heart sounds without murmurs. The abdomen was globose due to adipose panniculus, with visible venous collaterals but no organomegaly or tenderness. Examination of the extremities revealed varicose veins consistent with chronic peripheral venous insufficiency, without edema. 

Initial laboratory investigations revealed mild anemia (hemoglobin 11.6 g/dL) and atherogenic dyslipidemia, characterized by elevated triglycerides (155.6 mg/dL) and low HDL cholesterol (26.1 mg/dL). Other hematologic, renal, electrolyte, thyroid, and coagulation parameters were within normal limits (Table 2). 

**Table 2:** Initial laboratory results with reference ranges aPTT: Activated partial thromboplastin time; INR: international normalized ratio

Parameter	Result	Reference range
White blood cells (WBC, /µL)	4700	4,000 – 11,000
Neutrophils (/µL)	3619	1,800 – 7,500
Hemoglobin (g/dL)	11.6	12.0 – 16.0 (female)
Hematocrit (%)	35.9	36 – 46 (female)
Platelets (×10^3/µL)	266	150 – 450
Glucose (mg/dL)	77.5	70 – 100 (fasting)
Urea (mg/dL)	25.23	15 – 40
Creatinine (mg/dL)	0.92	0.6 – 1.1 (female)
Total cholesterol (mg/dL)	176	<200
Triglycerides (mg/dL)	155.6	<150
HDL cholesterol (mg/dL)	26.1	>50 (female)
LDL cholesterol (mg/dL)	118.8	<130 (optimal <100)
VLDL (mg/dL)	31.1	5 – 40
Sodium (mEq/L)	140	135 – 145
Potassium (mEq/L)	3.5	3.5 – 5.0
Chloride (mEq/L)	103	98 – 106
TSH (µIU/mL)	2.270	0.4 – 4.0
Free T4 (ng/dL)	1.480	0.8 – 1.8
Prothrombin time (s)	13.6	11 – 15
aPTT (s)	36.3	25 – 37
INR	1.01	0.8 – 1.2

**Figure 3:**
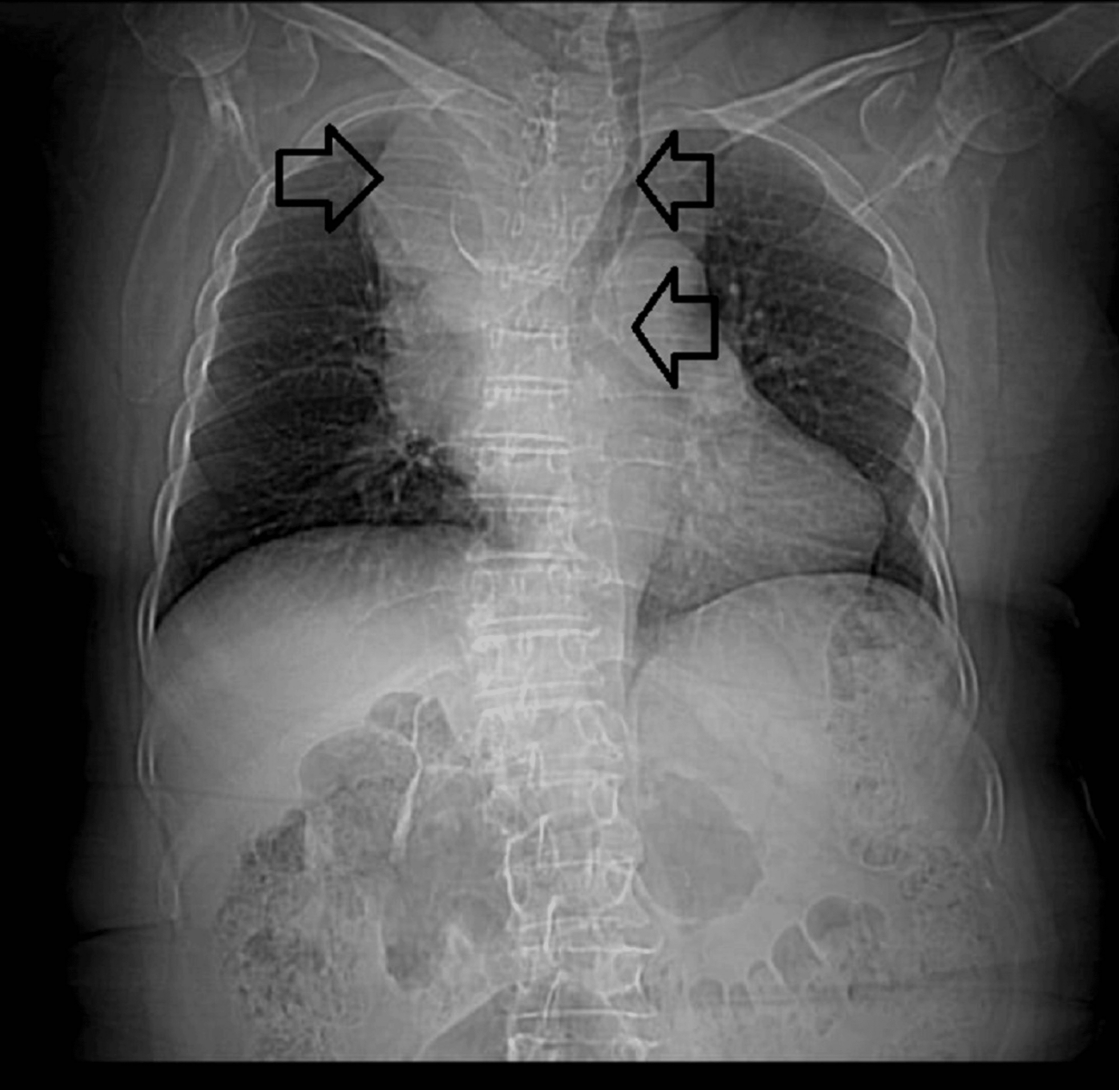
Non-contrast chest CT (coronal view) showing a bilobulated mass located in the anterior–superior mediastinum, causing compression of the superior vena cava and the brachiocephalic trunk, with rightward displacement of mediastinal structures and associated opacification, consistent with chronic superior vena cava obstruction

Chest computed tomography with and without intravenous contrast revealed a mediastinal mass in the anterior and superior compartments, bilobulated and containing calcifications within its wall (Figure 3). 

**Figure 4:**
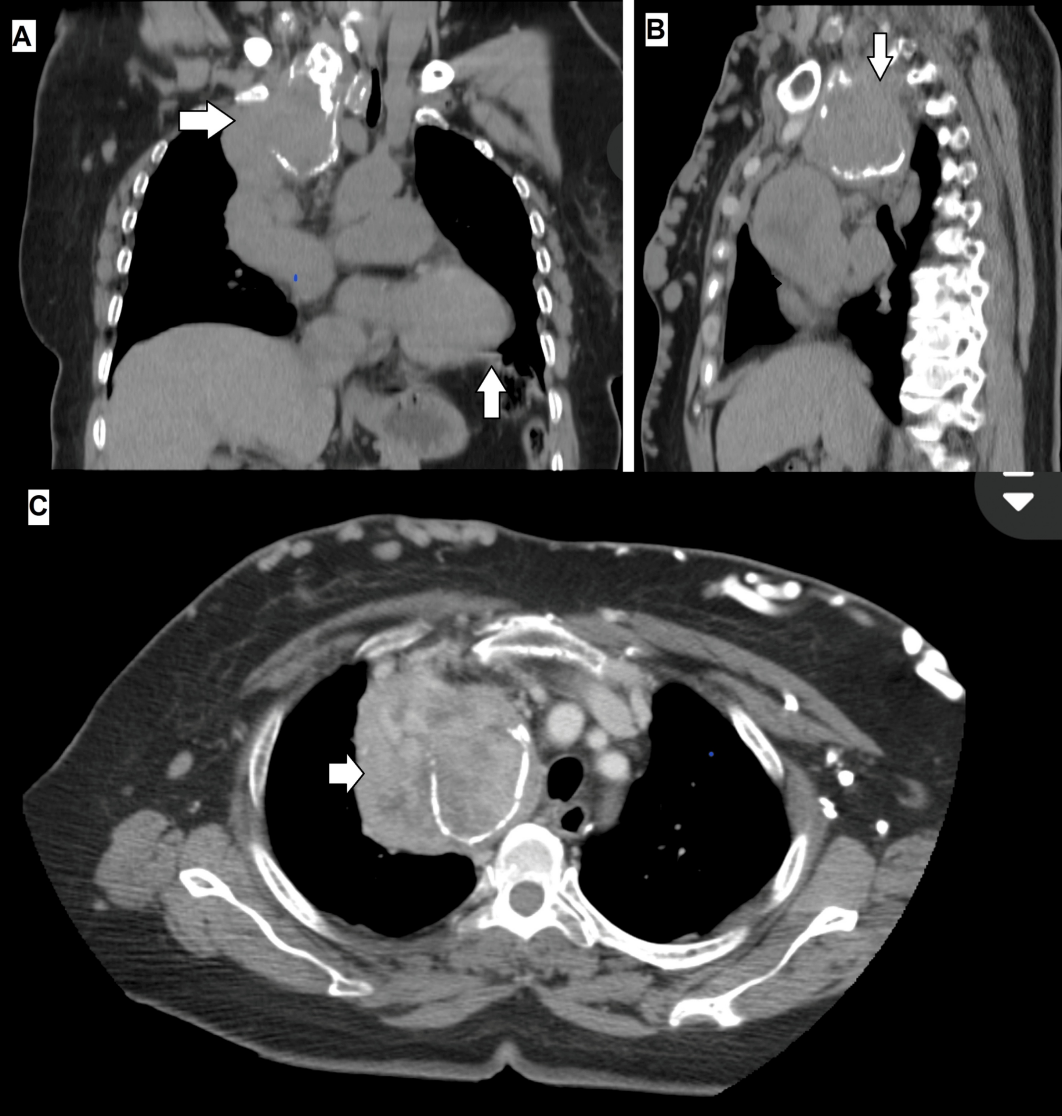
Contrast-enhanced chest CT (A) Coronal view showing a bilobulated anterior mediastinal mass compressing the right lung apex. (B) Sagittal view demonstrating severe compression of the superior vena cava and brachiocephalic trunk. (C) Axial view highlighting the mediastinal mass with heterogeneous density and wall calcifications, causing marked collateral venous dilatation.

The lesion measured approximately 70 mm anteroposterior, 67 mm transverse, and 121 mm longitudinal. It caused significant compression of the right lung, the superior vena cava, and the brachiocephalic trunk, resulting in marked dilatation of superficial venous collaterals. The mass showed heterogeneous density, with a peripheral component measuring 91 Hounsfield units (HU) on contrast-enhanced imaging and a central component of lower density (55 HU). No evidence of parenchymal pulmonary nodules, calcifications, or pleural effusion was noted. Cardiac cavities were preserved. The imaging impression favored the differential diagnosis of thymoma, lymphoma, or germ cell tumor (Figures 4A-4C). 

Subsequently, an ultrasound-guided biopsy of the mediastinal mass was performed. Cytological and histopathological evaluation revealed a hypocellular background with scant tubulo-glandular formations, composed of small cells with hyperchromatic nuclei, mild atypia without pleomorphism, and eosinophilic cytoplasm, highly suspicious for malignancy. The findings were most consistent with adenocarcinoma; however, immunohistochemical studies were not performed due to economic constraints, precluding definitive classification of the tumor’s lineage or origin. Given the anterior-superior mediastinal location, the presence of calcifications, and the prolonged, indolent course, the lesion is most plausibly a primary mediastinal germ cell tumor that underwent secondary somatic-type malignant transformation into adenocarcinoma. This mechanism, though uncommon, has been described in teratomatous germ cell tumors of the mediastinum and accounts for the coexistence of chronic evolution with malignant histology. A comprehensive immunohistochemical and systemic evaluation would be essential to confirm this diagnosis.

## Discussion

SVCS is classically described as an acute or subacute oncologic emergency, most frequently associated with lung cancer and lymphomas. In the scoping review by Wright et al., approximately 65-75% of malignant SVCS cases were secondary to lung cancer, and in up to 60% of cases, it represented the first manifestation of the underlying malignancy [6]. Clinical manifestations usually progress over weeks to months and include facial or neck edema, dyspnea, distended neck and thoracic veins, and upper extremity swelling [7]. In contrast, the patient described in our report remained compensated for over two decades, with extensive collateral venous circulation serving as the main clinical finding. Rather than reflecting a truly indolent tumor biology, this prolonged course likely resulted from the gradual development of collateral pathways that effectively mitigated venous congestion and delayed clinical recognition of the underlying malignant process. This case therefore underscores the remarkable adaptive capacity of the venous system and highlights the potential for misinterpreting slow-onset venous obstruction as a benign condition when symptoms remain subtle.

The case reported by Oh et al. illustrates the more typical presentation of malignant SVCS, with an 84-year-old man developing acute facial swelling within one week, secondary to small-cell lung carcinoma [8]. Chemotherapy resulted in rapid regression of symptoms and collateral vessels within days. Compared with that case, our patient’s decades-long evolution without respiratory compromise or neurological symptoms represents an atypical and rare clinical scenario.

From a therapeutic perspective, contemporary literature emphasizes endovascular stenting as an effective first-line or adjunctive intervention for rapid symptomatic relief, with technical success rates above 95% and symptomatic improvement within 48-72 hours. Chow et al. highlight that in cases of severe or life-threatening obstruction, such as with airway compromise or cerebral edema, stenting should be prioritized even before histologic confirmation, as it offers immediate palliation [9]. In contrast, when patients remain hemodynamically stable and well-compensated, as in our case, it is reasonable to prioritize obtaining a histologic diagnosis before intervention. This tailored approach allows clinicians to balance urgent decompression against diagnostic accuracy, ensuring individualized management based on symptom severity and collateral adaptation. In our patient, the prolonged subclinical course obviated the need for urgent decompression, permitting a complete diagnostic workup and histopathological confirmation of adenocarcinoma.

While primary mediastinal tumors are generally considered aggressive, the chronic evolution observed in our patient suggests an atypical biological behavior. Although a primary mediastinal germ cell tumor with secondary adenocarcinomatous transformation remains the most plausible explanation, the possibility of a primary thymic adenocarcinoma or malignant transformation of a pre-existing thymic epithelial lesion cannot be excluded. Maghbool et al. described such cases in their immunohistochemical and molecular study of thymic adenocarcinoma, reporting frequent expression of CK7, CK20, CDX2, villin, and MOC31, as well as genetic alterations involving chromosome 6p21.32, supporting the recognition of thymic adenocarcinoma as a distinct but exceptionally rare entity [9,10]. In our case, immunohistochemical testing could not be performed due to economic limitations, and although such evaluation was planned postoperatively, the patient elected to discontinue follow-up for personal reasons, representing a diagnostic limitation. This adaptive pathophysiological process explains why the patient remained asymptomatic for years and only sought medical attention for aesthetic concerns related to collateral venous engorgement, contrasting with the acute hemodynamic and respiratory compromise typically described in malignant SVCS [6].

Taken together, this case enriches current literature by illustrating a highly atypical presentation of malignant SVCS. While most reports emphasize rapid onset and urgent intervention, our findings emphasize the importance of maintaining a high index of suspicion in patients with chronic collateral venous circulation, even in the absence of acute symptoms. Moreover, this case highlights the continued value of clinical examination in an era dominated by imaging, as recognition of superficial venous patterns was pivotal in guiding the diagnostic pathway.

## Conclusions

This case illustrates an atypical and chronic presentation of SVCS, with more than two decades of progressive collateral venous circulation before definitive evaluation. Unlike most malignant SVCS cases, which typically present acutely and require urgent management, our patient remained clinically stable due to gradual venous adaptation. Histopathological confirmation revealed adenocarcinoma, underscoring the malignant etiology despite the indolent course. Chronic SVCS is rare but clinically relevant, as slow obstruction of the superior vena cava may allow long-term compensation and delay diagnosis. Collateral venous circulation represents a valuable diagnostic clue, and recognition of abnormal venous networks in the thoracic and abdominal wall should prompt investigation for central venous obstruction even in the absence of acute symptoms. Clinicians should be reminded that chronic, asymptomatic venous collaterals warrant thorough evaluation for potential malignancy, as early detection can alter prognosis and prevent severe complications. Comprehensive diagnostic evaluation remains essential, since establishing a histological diagnosis is critical to guide therapy and prognosis. This case highlights the importance of careful physical examination and the need for clinicians to consider malignant etiologies in patients with longstanding collateral venous circulation. Awareness of such atypical presentations can improve recognition, optimize diagnostic strategies, and ultimately influence patient outcomes.
